# The Effect of Nitrogen and Glyphosate on Survival and Colonisation of Perennial Grass Species in an Agro-Ecosystem: Does the Relative Importance of Survival Decrease with Competitive Ability?

**DOI:** 10.1371/journal.pone.0060992

**Published:** 2013-04-11

**Authors:** Christian Damgaard, Beate Strandberg, Solvejg K. Mathiassen, Per Kudsk

**Affiliations:** 1 Department of Bioscience, Aarhus University, Vejlsøvej 25, Silkeborg, Denmark; 2 Department of Agroecology, Aarhus University, Flakkebjerg, Slagelse, Denmark; USDA-ARS, United States of America

## Abstract

The ecological success of a plant species is typically described by the observed change in plant abundance or cover, but in order to more fully understand the fundamental plant ecological processes, it is necessary to inspect the underlying processes of survival and colonization and how they are affected by environmental conditions. A general ecological hypothesis on the effect of environmental gradients on demographic parameters is proposed and tested. The hypothesis is that decreasing fitness or competitive ability along an environmental gradient is associated with an increasing importance of survival for regulating the abundance of the species. The tested hypothesis is related to both the stress gradient hypothesis and whether the importance of competition increases along productivity gradients. The combined effect of nitrogen and glyphosate on the survival and colonization probability of two perennial grass species, *Festuca ovina* and *Agrostis capillaris*, which are known to differ in their responses to both glyphosate and nitrogen treatments, is calculated using pin-point cover data in permanent frames. We found that the relative importance of survival increased with the level of glyphosate for the glyphosate sensitive *A. capillaris* and decreased for the glyphosate tolerant *F. ovina*. Likewise, increasing levels of nitrogen increased the importance of survival for the relative nitrophobic *F. ovina*. Consequently, the proposed hypothesis was corroborated in this specific study. The proposed method will enable predictions of the effects of agricultural practices on community dynamics in a relatively simple setup eliminating the need to quantify all the interaction among the species in the plant community. The method will be immediately useful for the regulation of non-cultivated buffer strips between agricultural fields and semi-natural and natural biotopes such as hedgerows and waterways.

## Introduction

Biodiversity within European agricultural areas is declining due to the intensification of the agricultural practices [1]–[5]. More specifically, the repeated application of fertilizers and pesticide usage are generally regarded to affect the botanical composition in small natural and semi-natural biotopes in the neighbourhood of agricultural fields (see references in [6]). This is unfortunate since these biotopes play an important role in maintaining biodiversity in the agro-ecosystem by providing key habitats for flora and fauna, dispersal corridors, and they offer an important source of ecosystem services such as pollination [7]–[10]. Consequently, in order to predict effects of agricultural practices on the ecosystem dynamics in the neighbouring natural and semi-natural biotopes, it is of interest to study how fertilizer and herbicides affect the ecological success of different plant species commonly found in the agro-ecosystem.

Substantive and increasing knowledge exists on the effects of applying either fertilizers or herbicides on the biodiversity of higher plants in the agro-ecosystem, but studies of the combined effect of fertilizers and herbicide drift on non-target vegetation are still scarce (see references in [6]), and as a response to this important knowledge gap a replicated long-time field experiment sown with a mixture 31 grassland species and treated with various rates of nitrogen and glyphosate was set-up in 2001 [6]. In the long-term field experiment, the vegetation has gradually changed over the years both in respect to species richness and species composition. Generally, application of nitrogen as well as glyphosate has affected species richness negatively. However, at the highest nitrogen level (100 kg N/ha), the application of low dosages of glyphosate to some extent counteracts the effect of nitrogen on species richness. Regardless of the treatment, grasses dominate the vegetation at the experimental field, and the three grasses, common bentgrass (*Agrostis capillaris*), sheep's fescue (*Festuca ovina*) and couch grass (*Elytrigia repens*) make up the main part of the vegetation, but the composition of the grass community depends on the treatment. Previously, it has been documented that both nitrogen and glyphosate affected the competitive interactions of *F. ovina* and *A. capillaris* [6], [11], and the competitive effect of *F. ovina* increased with glyphosate [12]. Generally, *A. capillaris* performed best at low and intermediate glyphosate and nitrogen concentrations, although it seemed to be sensitive to competition from both *F. ovina* and *E. repens*.

The ecological success of a plant species is typically described by the observed change in plant abundance or cover, but in order to more fully understand the fundamental plant ecological processes leading to a change in plant abundance and make ecological predictions of the effect of environmental drivers, it is necessary to investigate and quantify the underlying ecological processes. In principle, this means that all the different interactions between the species in the plant community need to be investigated [13], but this is typically an exceedingly demanding task in multi-species plant communities, and instead we investigate how the vital rates of selected key species are affected by the environmental gradients [14].

Here, we will examine the combined effect of nitrogen and glyphosate on the survival and colonization probability of the two perennial grass species *Festuca ovina* and *Agrostis capillaris*, which are known to differ in their responses to both glyphosate and nitrogen treatments as summarized above. The underlying ecological hypothesis tested in the study is that decreasing fitness or competitive ability of a specific species along an environmental gradient is associated with an increasing importance of survival for regulating the abundance of the species. More specifically, we expect that the importance of survival relative to colonization increases with glyphosate dose for the glyphosate sensitive *A. capillaris* and decreases with glyphosate dose for the glyphosate tolerant *F. ovina*. Furthermore, we expect that that the importance of colonization relative to survival increases with nitrogen for the relative nitrophilous *A. capillaris*, while the opposite will be the case for *F. ovina*. The tested hypothesis is motivated by the notion that in a stressed environment, the resources that may be allocated to reproduction and clonal growth are limited and, consequently, the change in abundance will mainly be regulated by differential survival.

The tested hypothesis is related to both i) the stress gradient hypothesis [15], which predicts that the frequency of facilitative and competitive interactions will vary inversely across abiotic stress gradients, with facilitation being more common in conditions of high abiotic stress relative to more benign abiotic conditions, as well as to ii) the long-standing discussion in plant ecology, known as the Grime-Tilman debate, whether the importance of competition increases along productivity gradients [16], [17].

Typically, colonization and survival probabilities are estimated from demographic data of individual plants. However, in many natural plant communities dominated by perennial plants, e.g. grasslands, it is often difficult to distinguish individual plants due to their vegetative growth pattern and, consequently, to obtain reliable demographic data. Instead, it is possible to measure “colonization” and “survival” by considering the turnover of a species at a specific spatial point [18]. Considering such a specific spatial point, then if the species was present at time *t* but absent at time *t*+1, we may loosely speak of a mortality event, and if the species is absent from a specific pin-position at time *t* and present at time *t*+1, we may loosely speak of a colonization event [18]. We have chosen to use the term “colonization” rather than “recruitment”, since the event is defined by a novel occurrence in space [19], [20]. However, in the interpretation of the results it is important to remember that the concepts of colonization and mortality have a different meaning than usual in studies where individuals are considered. For perennial plant species that spread clonally by forming well-defined ramets, the concepts of colonization and mortality at a certain pin position make apparent biological sense, whereas for more plastic species with variable sizes, the concepts are inadequate descriptions of the underlying biological causes of a change in plant abundance. Bearing this issue of terminology in mind, we may, generally, assert that the cover of a plant species will increase with colonization and decrease with mortality.

## Materials and Methods

### Field experiment

The field experiment was designed to study the ecological processes, including establishment, survival and competitive interactions, of a semi-natural ecosystems affected by herbicide (glyphosate) and fertilizer (nitrogen) in a relatively realistic way.

The selected area was a former agricultural field on dry, nutrient poor sandy soil in Djursland, Denmark. The field laid fallow for a couple of years prior to the start of the experiment in 2001. The field is quadrangular and surrounded by forest on two sides (south and west) and separated from the neighbouring fields by 5 meter broad hedgerows on the other sides.

In 2001, the area was deep ploughed down to 60 cm to minimize establishment from the soil seed bank and prepared for the experiment by harrowing and rolling. Thirty-one selected grassland plant species covering different life form strategies (CRS strategies sensu [21]) were sown in spring 2001 [22]. Since 2001, the area has been undisturbed except for the application of the experimental treatments and removal of woody species (trees and bushes) every year prior to herbicide application.

No specific permits were required for the described field studies. The area is a privately own former agricultural field that has been rented on a long-time basis with the sole purpose of doing scientific experiments.

### Treatments

The experimental manipulations were set up as a completely randomized block design with 10 replicates of twelve treatments. Each replicate plot was 7 m×7 m with a buffer zone of 1.5 m surrounding the plot. A buffer zone of 10 m separated the experiment from the surrounding vegetation. The buffer zones were also sown with the seed mixture.

The treatments included 4 glyphosate treatments (0; 14.4; 72 and 360 g a.i./ha RoundupBio®, Monsanto Crop Science, Denmark A/S, 360 g/L glyphosate as a isopropylamine salt) and 3 nitrogen treatments (0, 25 and 100 kg N/ha) applied in a full factorial design. The applied glyphosate doses were equal to 0, 1, 5 and 25%, respectively, of the dose recommended for pre- and post-harvest treatment against perennial weeds, which is the most common use of glyphosate in Denmark [23]. All 120 plots received phosphorus (53 kg/ha), potassium (141 kg/ha), sulphur (50 kg/ha) and copper (0.7 kg/ha) every year.

For the herbicide applications, experimental spraying equipment was used. The boom was fitted with Lurmark Low-drift LD 015 Green nozzles operated at a pressure of 2.0 bars delivering a spray volume of 300 L/ha. The wind speed on the days selected for spraying was very low (0–2 m/s). It did not rain during the days following the application. The amount of fertilizer was weighed individually for each plot and spread by hand. The plots were treated by glyphosate for the first time on 24 August 2001. Since then, glyphosate and fertilizer treatments were carried out once every year in mid-May [11].

### Plant species

*F. ovina* and *A. capillaris* are perennial grasses. They both have a caespitose growth form, but the turfs formed by *F. ovina* generally are much denser than those formed by *A. capillaris*. In contrast to *A. capillaris* which is deciduous, *F. ovina* is winter green and productive through the winter as long as temperatures are above zero. Furthermore, *F. ovina* has narrow, curled and waxy leaves, whereas *A. capillaris* has broader, flat leaves without any wax cover.

*F. ovina* has previously been shown to be more tolerant to glyphosate than *A. capillaris* [11], [24], whereas *A. capillaris* is a more nitrophilous species than *F. ovina* (the Ellenberg N values [25] of the two species are 4 and 1, respectively)

### Sampling

In order to study the effect of nitrogen and glyphosate on *F. ovina* and *A. capillaris*, one permanent 0.5 m×0.5 m quadrate was placed within each of the 120 plots in 2007. The quadrate was not placed randomly, but in such a way that both *F. ovina* and *A. capillaris* were noticeably abundant in the quadrate. Local presence-absence data of the two species were determined by the pin-point method [26], [27] using a pin-point frame with the same dimension as the quadrate. The frame had 25 pin-positions regularly placed at a distance of 10 cm. At each position, a sharply pointed pin with a diameter of 0.5 mm was passed vertically through the vegetation and presence-absence data of the two species were recorded for each pin.

The sampling was performed three times a year for three years in the period 2007–2009 [6], but in this analysis, the sampling that was made in the spring approximately two weeks after the herbicide application was used.

### Model

The quantitative effect of survival and colonization processes on population growth of individuals is most effectively summarized using population models [28], and the calculation of elasticity (the relative contribution of different demographic parameters on population growth rates) has particularly been shown to be a powerful tool to investigate the importance of different demographic variables in determining population growth [18].

If absence-presence data of species *A* from two successive recordings from the same pin-position are considered, there are four possible transition events and corresponding probabilities (Table 1). These transition probabilities depend on i) the probability (*p*) that a plant of species *A* is present at time *t*, ii) survival; the probability (*s*) that a plant of species *A* is present at the pin-position at both time *t* and time *t*+1, and iii) colonization; the probability (*c*) that a plant of species *A* is present at the pin-position at time *t*+1 but was absent at time *t* [18]. The possibility of a combined mortality and colonization events is ignored.

**Table 1:** The four possible events of two successive recordings of presence absence data and their corresponding probabilities, *p*: current probability, *c*: colonization probability, *s*: survival probability.

Event	Description	Probability 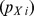
X_1_: *A_t_*, *A_t_*_+1_	A plant of species A was present in year *t* and was also present in year *t*+1	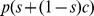
X_2_: *nA_t_*, *nA_t_*_+1_	A plant of species A was not present in year *t* and was also not present in year *t*+1	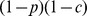
X_3_: *A_t_*, *nA_t_*_+1_	A plant of species A was present in year *t* but was not present in year *t*+1 (indicates a possibly event of mortality)	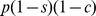
X_4_: *nA*_t_, *A_t_*_+1_	A plant of species A was not present in year *t* but was present in year *t*+1 (indicates a possibly event of colonization)	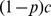

The change in the probability that species *A* is present in year *t* to *t*+1 is denoted by *π* and may be defined, analogous to the population growth rate *λ* of individuals [28], as the ratio between the probability 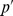 that species *A* is present at time *t*+1 and the probability *p* that species *A* is present at time *t* :
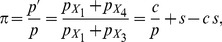
(1)where 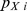 are defined in Table 1.

**Figure 1:**
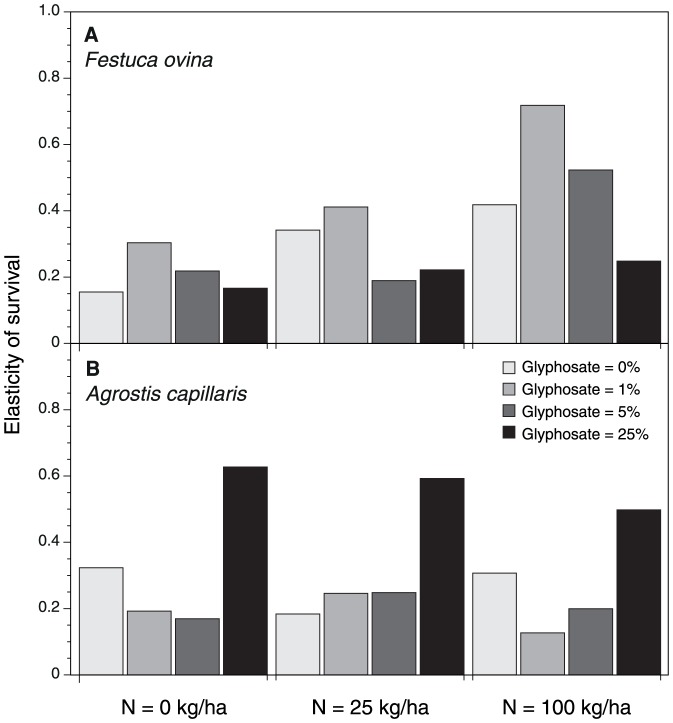
The elasticity of the survival probability of *Festuca ovina* (a) and *Agrostis capillaris* (b) at different treatment levels of nitrogen and glyphosate.

From the above definition it is apparent that if 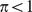, then the probability that species *A* is present decreases, and if 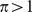, then the probability that species *A* is present increases. Furthermore, the change in the probability that species *A* is present is a function of the current probability (*p*), the colonization probability and the survival probability probabilities (Fig. 1). Note also that the plant cover of species *A* at time *t* is estimated by taking the mean of *p* across space.

The change in the probability of being present, as defined above in equation (1), is always positive, which ensures that it is possible to calculate both the sensitivity and elasticity of the change [28]. The sensitivity of the change in the probability that species *A* is present is a function of the colonization and survival probabilities which are defined as:
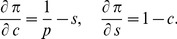
(2)


Likewise, the elasticity of the change in the probability that species *A* is present is a function of the colonization and survival probabilities which are defined as:
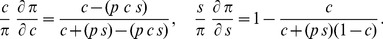
(3)


### Estimation and statistical inferences

The colonization and survival probabilities may be estimated from data of the four possible events of two successive recordings of presence-absence data 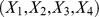, which are defined in Table 1, by maximizing the likelihood function of the multinomial distribution 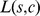 using the transition probabilities specified in Table 1 and assuming that the plants are distributed homogenously across the site of the investigation so that the probability that species *A* is present at time *t* 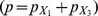 may be estimated as 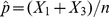:
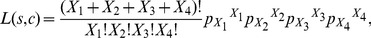
(4)Different hypotheses on the treatment effect on the maximum likelihood estimates of elasticity of survival probabilities on cover change were tested using linear models where the residual error was assumed to be normally distributed (this assumption was checked using residual plots). Only the elasticity of the survival probability was analyzed, since the elasticity of colonization probability, due to the mathematical definition of elasticities (3), showed similar but opposite results than the elasticity of survival probability.

## Results

The maximum likelihood estimates as well as the sensitivity and elasticity of the survival and colonization probability of the two grass species *F. ovina* and *A. capillaris* at different treatment levels of nitrogen and glyphosate are shown in Table 2 and 3 for both yearly changes and all years together. However, the main findings of the study are most easily communicated by the calculated elasticity of one of the complementing demographic parameters. Thus, the effect of nitrogen and glyphosate on the survival elasticity (the relative contribution of the survival probability of the change in regulating plant cover) is shown in Fig. 1 for both species for all years and analyzed using linear models (Table 4 and 5).

**Table 2:** The maximum likelihood estimates as well as the sensitivity and elasticity of the survival (s) and colonization (c) probability of *Festuca ovina* at different treatment levels of nitrogen and glyphosate.

Nitrogen	Glyphosate	Year	s	c	*p*	Sen. s	Sens. c	Elast. s	Elast. c
0	0	2007–2008	0.427	0.422	0.906	0.578	1.136	0.272	0.529
0	0	2008–2009	0.610	0.752	1.448	0.248	1.114	0.104	0.579
0	0	All	0.451	0.600	-	0.400	1.189	0.155	0.613
0	1	2007–2008	0.588	0.293	0.963	0.707	1.277	0.432	0.389
0	1	2008–2009	0.642	0.545	1.349	0.455	1.296	0.216	0.524
0	1	All	0.605	0.422	-	0.578	1.296	0.304	0.475
0	5	2007–2008	0.543	0.371	0.948	0.629	1.091	0.360	0.427
0	5	2008–2009	0.561	0.686	1.359	0.314	1.163	0.130	0.587
0	5	All	0.538	0.535	-	0.465	1.139	0.218	0.531
0	25	2007–2008	0.410	0.441	0.835	0.559	0.964	0.274	0.509
0	25	2008–2009	0.429	0.643	1.211	0.357	1.216	0.127	0.646
0	25	All	0.380	0.560	-	0.440	1.117	0.166	0.622
25	0	2007–2008	0.537	0.231	0.808	0.769	1.175	0.511	0.336
25	0	2008–2009	0.692	0.477	1.373	0.523	1.427	0.263	0.496
25	0	All	0.574	0.369	-	0.631	1.320	0.342	0.459
25	1	2007–2008	0.674	0.237	0.902	0.763	0.960	0.570	0.252
25	1	2008–2009	0.762	0.482	1.268	0.518	1.049	0.311	0.399
25	1	All	0.701	0.368	-	0.632	1.017	0.411	0.349
25	5	2007–2008	0.449	0.432	0.869	0.568	0.971	0.293	0.483
25	5	2008–2009	0.812	0.722	1.405	0.278	0.822	0.161	0.422
25	5	All	0.525	0.596	-	0.404	0.994	0.190	0.530
25	25	2007–2008	0.627	0.483	0.953	0.517	0.675	0.340	0.342
25	25	2008–2009	0.748	0.761	1.219	0.239	0.618	0.147	0.386
25	25	All	0.652	0.632	-	0.368	0.681	0.222	0.398
100	0	2007–2008	0.509	0.126	1.257	0.874	5.919	0.354	0.595
100	0	2008–2009	0.528	0.092	0.976	0.908	4.853	0.491	0.459
100	0	All	0.519	0.110	-	0.890	5.340	0.418	0.530
100	1	2007–2008	0.391	0.005	0.409	0.995	3.397	0.950	0.045
100	1	2008–2009	0.764	0.058	1.259	0.942	8.495	0.571	0.393
100	1	All	0.488	0.034	-	0.966	4.889	0.718	0.256
100	5	2007–2008	0.651	0.178	0.922	0.822	1.523	0.581	0.294
100	5	2008–2009	0.535	0.208	0.915	0.792	1.823	0.463	0.415
100	5	All	0.596	0.194	-	0.806	1.666	0.523	0.351
100	25	2007–2008	0.471	0.385	0.849	0.615	0.983	0.341	0.445
100	25	2008–2009	0.782	0.618	1.354	0.382	0.926	0.221	0.422
100	25	All	0.555	0.517	-	0.483	1.016	0.248	0.486

**Table 3:** The maximum likelihood estimates as well as the sensitivity and elasticity of the survival (s) and colonization (c) probability of *Agrostis capillaris* at different treatment levels of nitrogen and glyphosate.

Nitrogen	Glyphosate	Year	s	c	*p*	Sen. s	Sens. c	Elast. s	Elast. c
0	0	2007–2008	0.179	0.072	0.595	0.928	5.774	0.279	0.699
0	0	2008–2009	0.519	0.084	1.320	0.916	9.481	0.360	0.607
0	0	All	0.304	0.079	-	0.921	7.159	0.323	0.649
0	1	2007–2008	0.087	0.144	0.727	0.856	4.458	0.103	0.880
0	1	2008–2009	0.319	0.119	1.025	0.881	5.931	0.274	0.689
0	1	All	0.189	0.131	-	0.869	5.075	0.192	0.779
0	5	2007–2008	0.199	0.108	0.661	0.892	4.265	0.269	0.699
0	5	2008–2009	0.071	0.127	0.919	0.873	6.685	0.068	0.922
0	5	All	0.147	0.118	-	0.882	5.230	0.169	0.808
0	25	2007–2008	0.057	0.005	0.082	0.995	5.046	0.689	0.308
0	25	2008–2009	0.247	0.004	0.500	0.996	62.253	0.492	0.506
0	25	All	0.071	0.004	-	0.996	9.363	0.627	0.370
25	0	2007–2008	0.101	0.082	0.350	0.918	3.024	0.265	0.712
25	0	2008–2009	0.067	0.081	0.786	0.919	8.861	0.079	0.914
25	0	All	0.093	0.082	-	0.918	4.537	0.184	0.800
25	1	2007–2008	0.242	0.129	0.524	0.871	2.185	0.402	0.539
25	1	2008–2009	0.227	0.209	1.148	0.791	4.402	0.157	0.802
25	1	All	0.220	0.175	-	0.825	2.964	0.246	0.702
25	5	2007–2008	0.157	0.093	0.485	0.907	3.520	0.292	0.678
25	5	2008–2009	0.015	0.046	0.364	0.954	7.561	0.040	0.958
25	5	All	0.119	0.068	-	0.932	4.832	0.248	0.734
25	25	2007–2008	0.102	0.010	0.148	0.990	4.528	0.681	0.312
25	25	2008–2009	0.367	0.012	0.750	0.988	30.883	0.483	0.510
25	25	All	0.135	0.011	-	0.989	7.929	0.592	0.401
100	0	2007–2008	0.125	0.037	0.342	0.963	5.796	0.352	0.634
100	0	2008–2009	0.148	0.033	0.588	0.967	13.146	0.243	0.749
100	0	All	0.133	0.035	-	0.965	8.060	0.307	0.682
100	1	2007–2008	0.041	0.014	0.135	0.986	6.716	0.296	0.700
100	1	2008–2009	0.000	0.020	1.000	0.980	50.000	0.000	1.000
100	1	All	0.031	0.017	-	0.983	11.874	0.127	0.871
100	5	2007–2008	0.029	0.047	0.175	0.953	3.096	0.160	0.832
100	5	2008–2009	0.051	0.021	0.429	0.979	17.806	0.117	0.880
100	5	All	0.044	0.032	-	0.968	5.275	0.200	0.794
100	25	2007–2008	0.092	0.000	0.092	1.000	3.197	1.000	0.000
100	25	2008–2009	0.000	0.020	0.714	0.980	35.143	0.000	1.000
100	25	All	0.073	0.012	-	0.988	5.943	0.498	0.496

**Table 4:** Analysis of variance of the elasticity of the survival probability on the change in the cover of *Festuca ovina* for all years.

	DF	SS	MS	F-Statistic	P-Value
Nitrogen	1	0.1487	0.1487	14.4757	0.0052
Glyphosate	1	0.0566	0.0566	5.5079	0.0469
Glyphosate * Nitrogen	1	0.0237	0.0237	2.3059	0.1674
Error	8	0.0822	0.0103		
Total	11	0.3111			

Fitted model: 0.236+0.00342 Nitrogen−0.00248 Glyphosate−0.000103 Glyphosate * Nitrogen (adjusted r^2^: 64%).

**Table 5:** Analysis of variance of the elasticity of the survival probability on the change in the cover of *Agrostis capillaris* for all years.

	DF	SS	MS	F-Statistic	P-Value
Nitrogen	1	0.0045	0.0045	0.6797	0.4336
Glyphosate	1	0.2574	0.2574	39.0347	0.0002
Glyphosate * Nitrogen	1	0.0045	0.0045	0.6825	0.4327
Error	8	0.0527	0.0066		
Total	11	0.3191			

Fitted model: 0.202−0.000106 Nitrogen+0.0163 Glyphosate−0.000045 Glyphosate * Nitrogen (adjusted r^2^: 77%).

As expected, *F. ovina* and *A. capillaris* responded differently to the nitrogen and glyphosate treatments. When the level of glyphosate increased, survival became more important than colonization for regulating the cover of the glyphosate sensitive *A. capillaris* (Fig. 1a; Table 5: P = 0.0002), whereas survival tended to become less important for the glyphosate tolerant *F. ovina* (Fig. 1b; Table 4: P = 0.047). Increasing levels of nitrogen increased the importance of survival for regulating the cover of the relative nitrophobic *F. ovina* (Fig. 1a; Table 4: P = 0.0052), whereas there was no significant effect of nitrogen on the elasticity of survival for *A. capillaris* (Fig. 1b; Table 5: P = 0.43). There was no significant interaction effect of glyphosate and nitrogen on the elasticity of survival for the two investigated species.

## Discussion

As mentioned previously, the calculation of elasticity of different birth and death processes on population growth rates has been shown to be a powerful tool for investigating the importance of different demographic variables in determining population growth. More specifically, in this study the elasticity of survival has proven useful for quantifying the relative importance of survival for regulating plant cover along an environmental gradient.

The general hypothesis tested in the study, i.e. that decreasing fitness or competitive ability of a specific species along an environmental gradient is associated with an increasing importance of survival for regulating the abundance of the species (as measured by the elasticity of survival), was corroborated in the study. More specifically, the relative importance of survival increased with the level of glyphosate for the glyphosate sensitive *A. capillaris* and decreased for the glyphosate tolerant *F. ovina*. Likewise, increasing levels of nitrogen increased the importance of survival for the relative nitrophobic *F. ovina*. Naturally, the generality of the proposed hypothesis needs to be tested in other biological systems and along different environmental gradients.

As mentioned above, the tested hypothesis is related to both i) the stress gradient hypothesis [15], which predicts that the frequency of facilitative and competitive interactions will vary inversely across abiotic stress gradients, with facilitation being more common in conditions of high abiotic stress relative to more benign abiotic conditions, as well as to ii) the long-standing discussion in plant ecology, known as the Grime-Tilman debate, whether the importance of competition increases along productivity gradients [16], [17]. However, assigning the frequency or importance to the competitive interactions is not trivial and e.g. depends on the investigated life stage [15] and is, typically, confounded with the underlying stress or productivity gradient [29]. Consequently, in the cases where a limited experimental design precludes the parameterization of a full population ecological model, it may be more robust to focus on the underlying demographic parameters, e.g. survival and colonization, in investigations of environmental gradients instead of calculating the importance of competitive interactions.

It is important to remember that the estimated colonization and survival probabilities as well as the connected elasticities are calculated from repeated pin-point cover data and only are meaningful for plant species with a constant or modular type of growth. For plant species with individual size variation from year to year and with a variable spatial arrangement of tissues, the estimated variables will rather be a characterization of the growth form of the individual plant than an estimation of demographic parameters. Since grass species generally, including both *F. ovina* and *A. capillaris*, are relatively modular plant species, we think that the estimated parameters to a certain degree reflect the “population dynamic” of the ramets of the two grass species.

In many countries, legislation or incentives have been put in place to increase the area of non-cultivated buffer strips between agricultural fields and semi-natural and natural biotopes such as hedgerows and waterways. The empirical data available to determine e.g. the width of the buffer strips required to protect those biotopes is often inadequate, partly because it is resource demanding to conduct such experiments. Furthermore, it can be difficult to generalize on the basis of data originating from few sites. We propose an alternative approach making use of the model presented in this paper. If data were available on the susceptibility of the key species in the biotope in question and this information was combined with existing spray drift models (e.g. [30]), the impact of herbicides could be simulated for different widths of the buffer strips. If herbicide sensitivity data are not already available, they would be easy and relatively inexpensive to generate. Simulating effects of nitrogen would be more difficult because nitrogen transfer across a buffer strip is more complex and less understood than that of pesticides. Nonetheless, simple models describing nitrogen gradients across buffer strips could be developed and incorporated in the model. Besides predicting the combined effect of herbicide and nitrogen exposure on the species composition, the model could also provide insight into the relative impact of herbicides and nitrogen.
